# ﻿Two new species of *Microlicia* (Melastomataceae) from unprotected campo rupestre in the western Chapada Diamantina, Bahia, Brazil

**DOI:** 10.3897/phytokeys.266.158601

**Published:** 2025-11-19

**Authors:** Ricardo Pacifico, Frank Almeda, Mathias Erich Engels, Renato Goldenberg

**Affiliations:** 1 São Paulo State University (UNESP), Department of Biology, School of Agricultural and Veterinary Sciences, Jaboticabal, São Paulo, 14884-900, Brazil; 2 California Academy of Sciences, Institute for Biodiversity Science and Sustainability, Department of Botany, 55 Music Concourse Drive, Golden Gate Park, San Francisco, California 94118-4503, USA; 3 Universidade Federal do Paraná, Programa de Pós-graduação em Botânica, Cx. P. 19031, Jardim das Américas, Curitiba, Paraná, 81530-900, Brazil; 4 Universidade Federal do Paraná, Department of Botany, Campus do Centro Politécnico, R. Francisco H. dos Santos s.n., Curitiba, Paraná, 81531-980, Brazil

**Keywords:** Endemism, Ibitiara, Lavoisiereae, taxonomy

## Abstract

*Microlicia* D.Don (Melastomataceae, Lavoisiereae) is a genus of shrubs and subshrubs comprising nearly 300 species, primarily distributed across nutrient-poor, open habitats in Brazil’s campos rupestres, cerrado, and other montane ecosystems. During botanical surveys in previously unexplored campo rupestre areas in the municipality of Ibitiara (Bahia, Brazil), we discovered two undescribed species, herein named *Microlicia integra* and *Microlicia flavistyla*. The campo rupestre of Ibitiara is located at the westernmost limits of the Chapada Diamantina, a region known for its scenic landscapes and high levels of plant endemism. We provide formal descriptions, illustrations, photographs of living specimens, distribution maps, and notes on their taxonomy and conservation status. Our findings highlight the rich yet still insufficiently known floristic diversity of the unprotected campo rupestre areas of western Chapada Diamantina and underscore the urgent need for conservation measures in these fragile ecosystems.

## ﻿Introduction

*Microlicia* D.Don (Melastomataceae, Lavoisiereae) comprises nearly 300 species of shrubs and subshrubs that occur predominantly in open, nutrient-poor habitats across campo rupestre, cerrado, and other montane ecosystems in Brazil (Versiane et al. 2021; Pacifico and Almeda 2022a), with only 11 species growing in neighboring countries such as Bolivia, Colombia, Peru, Venezuela, and Guyana (Pacifico et al. 2020a; Versiane et al. 2020).

In its current delimitation, *Microlicia* can be distinguished from other genera of Lavoisiereae by its woody habit, diplostemonous flowers, and the absence of morphological features found in the other two genera of the tribe (Versiane et al. 2021; Pacifico and Almeda 2022a). *Rhynchanthera* DC., the first diverging lineage within Lavoisiereae, is notable for its haplostemonous flowers with a single whorl of fertile stamens and staminodia and conspicuously long anther rostra (usually >2 mm) (Renner 1990; Fritsch et al. 2004). In contrast, *Poteranthera* Bong., a genus of six species sister to *Microlicia* s.l., includes herbaceous annuals with ebracteolate apical flowers, leaves bearing stout glandular trichomes along the margin, a hypanthium constricted distally below the torus, and either haplostemonous or diplostemonous flowers (Kriebel 2012; Almeda and Pacifico 2018, 2023; Versiane et al. 2021).

During botanical surveys in campo rupestre sites of western Bahia, we collected specimens of *Microlicia* that could not be assigned to any known species. Our morphological analyses confirmed the existence of two undescribed taxa, discovered in a previously unexplored area of campo rupestre in the municipality of Ibitiara, at the westernmost limits of the Chapada Diamantina. This region, formed mainly by Proterozoic metasedimentary rocks (ca. 900 mya) that create striking landscapes of considerable touristic significance, is a prominent mountainous feature of central Bahia (northeastern Brazil). It is characterized by high plateaus ranging from 600 to 2033 m in elevation (Lima and Nolasco 2015).

The Chapada Diamantina is part of the northern sector of the Cadeia do Espinhaço, known for its rugged topography, quartzitic substrates, and distinctive flora with high levels of endemism (Giulietti et al. 1997). It is already recognized as one of the main areas of endemism for *Microlicia* (Pacifico et al. 2020b), with several new species recently described from the area, underscoring the importance of continued botanical exploration (see Gali et al. 2022; Pacifico and Almeda 2022b, 2022c; Pacifico et al. 2022a, 2022b; Romero et al. 2025). In this study, we provide detailed morphological descriptions of the new species, along with illustrations, photographs of living plants, distribution maps, and notes on their taxonomic affinities and conservation status.

## ﻿Materials and methods

This study is based on the examination of herbarium specimens housed at CAS, HUEFS, RB, SPF, and UPCB (herbarium acronyms follow Thiers 2025, continuously updated). Analyses included type specimens and representative collections of the taxa discussed herein, alongside original species descriptions (protologues). Comparative morphological characters of *Microlicia minima* Markgr. were obtained from Baumgratz et al. (1995) and Pataro et al. (2017), whereas characters of *M. oligochaeta* Wurdack, *M. pinheiroi* Wurdack, and *M. wurdackiana* Almeda & A.B.Martins were obtained from their respective protologues (Wurdack 1983; Almeda and Martins 2012). Geographic distribution maps were generated using QGIS version 3.4.6 (QGIS Development Team 2025), based on specimen label coordinates.

### ﻿Taxonomic treatment

#### ﻿ Microlicia integra

Taxon classificationPlantaeMyrtalesMelastomataceae

R.B.Pacifico, Almeda & R.Goldenb. sp. nov.

AA525571-A87D-5957-B941-D8C37692E3A6

urn:lsid:ipni.org:names:77372162-1

Figs 1, 2B, C

##### Type.

Brazil • Bahia: Ibitiara. Serra de Ibitiara, Chapada Diamantina, 12°35'50"S, 42°05'34.7"W, 1,218 m, 19 December 2024, fl. fr., *R. Pacifico 736 & I.V. Castro* (holotype: UPCB barcode UPCB0095366!, isotypes: CAS!, HUEFS!, RB!, SPF!).

**Figure 1.**
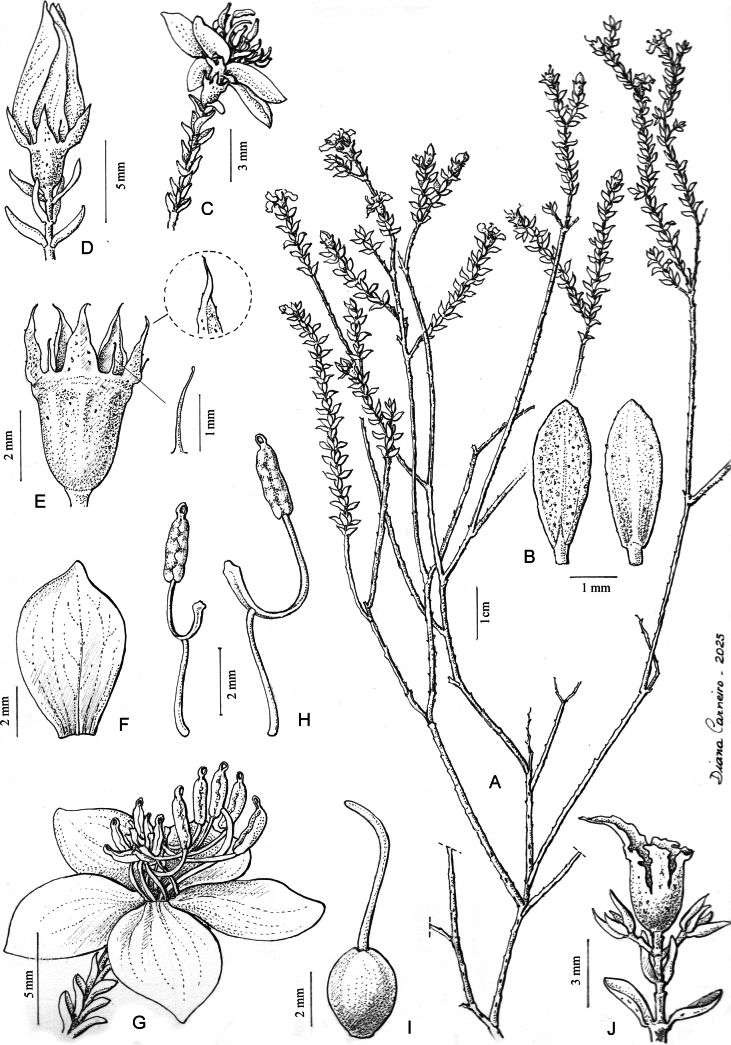
*Microlicia integra*. **A.** Habit; **B.** Leaf adaxial (left) and abaxial (right) surfaces; **C.** Branchlet terminating in a flower at anthesis; **D.** Apical branch terminating in a floral bud; **E.** Flowering hypanthium and calyx lobes; **F.** Petal in adaxial view; **G.** Flower in lateral view; **H.** Antepetalous (left) and antesepalous (right) stamens; **I.** Gynoecium; **J.** Branchlet terminating in a capsule enveloped by the hypanthium. Drawn from R. Pacifico 736 & I.V. Castro.

##### Diagnosis.

Differs from *M. minima* Markgr. by the petiolate leaves (vs. sessile in *M. minima*), each calyx lobe alternating with an eglandular trichome 0.7–1 mm long (vs. these trichomes absent), petals entirely pink (vs. magenta with a cream base) that are 7–11 mm long (vs. 4.5–6 mm long), and dimorphic stamens (vs. isomorphic to subisomorphic).

##### Description.

Copiously branched erect shrubs 0.3–1.3 m tall. Branchlets light green (when fresh), quadrangular, glandular-punctate, the stem angles with inconspicuous narrow wings ca. 0.1 mm wide. Leaves ascending (when fresh), flat, decussate, ca. 2 times longer than the internodes, on short flattened petioles 0.2–0.6 mm long; blades 1.8–3.9(–4.5) × 0.7–1.2(–1.6) mm, narrowly elliptic, papyraceous, both surfaces vivid green when fresh, pale green when dry, base cuneate to attenuate, margin entire, the apex acute to obtuse, 1-nerved from the base, venation slightly impressed on the abaxial surface and inconspicuous on the adaxial surface, both surfaces densely glandular-punctate. Flowers 5-merous on inconspicuous pedicels 0.3–0.4 mm long, solitary, terminal, not clustered at the apex of the branchlets. Hypanthia (at anthesis) 2.4–2.7 mm long, 2.5–3 mm wide at the torus, yellowish green when fresh, turning brown when dry, campanulate, densely glandular-punctate. Calyx tubes 0.1–0.3 mm long; calyx lobes (at anthesis) 1.6–2.0 mm long (excluding apical trichome), 0.9–1.3 mm wide at the base, triangular, apex acute terminating in an eglandular trichome ca. 0.4–0.5 mm long (tardily deciduous), externally glandular-punctate, the margin entire and glabrous, each calyx lobe alternating with an eglandular trichome ca. 0.7–1 mm long (tardily deciduous). Petals 7–11 × 5–7 mm, widely obovate, pink, margin entire, apex rounded to bluntly acute, both surfaces glabrous. Stamens 10, dimorphic; larger (antesepalous) stamens 5, filaments 3.6–4.3 mm long, yellow, pedoconnectives 6.1–6.9 mm long, yellow, appendages 1.3–1.6 mm long, yellow, apex truncate to slightly emarginate, thecae 1.9–2.1 mm long (excluding rostra), oblong, yellow, externally corrugated (polysporangiate), rostra 0.4–0.5 mm long, white, the circular pores 0.2–0.3 mm wide, ventrally inclined; smaller (antepetalous) stamens 5, filaments 3.2–3.8 mm long, yellow, pedoconnectives 2.9–3.3 mm long, yellow, appendages 0.9–1.1 mm long, apex truncate, thecae 1.3–1.6 mm long (excluding rostra), oblong, yellow becoming brownish in post-anthesis, externally corrugated (polysporangiate), rostra 0.3–0.5 mm long, the circular pores 0.1–0.2 mm wide, ventrally inclined. Ovaries ca. 1.6 × 1.3 mm, subglobose, superior, glabrous, 3-locular; styles 7.1–8.5 mm long, linear, pink with a yellow base, stigma punctiform. Loculicidal capsules 2.7–2.9 × 2.3–2.7 mm (immature), ovoid, brownish when dry, initially enveloped by the ± constricted hypanthium at the apex, then tardily rupturing and flaking away with age, the apex not exceeding the torus, dehiscent from the apex to the base, columellas caducous. Seeds not seen.

##### Distribution, habitat, and phenology.

**Figure 2.**
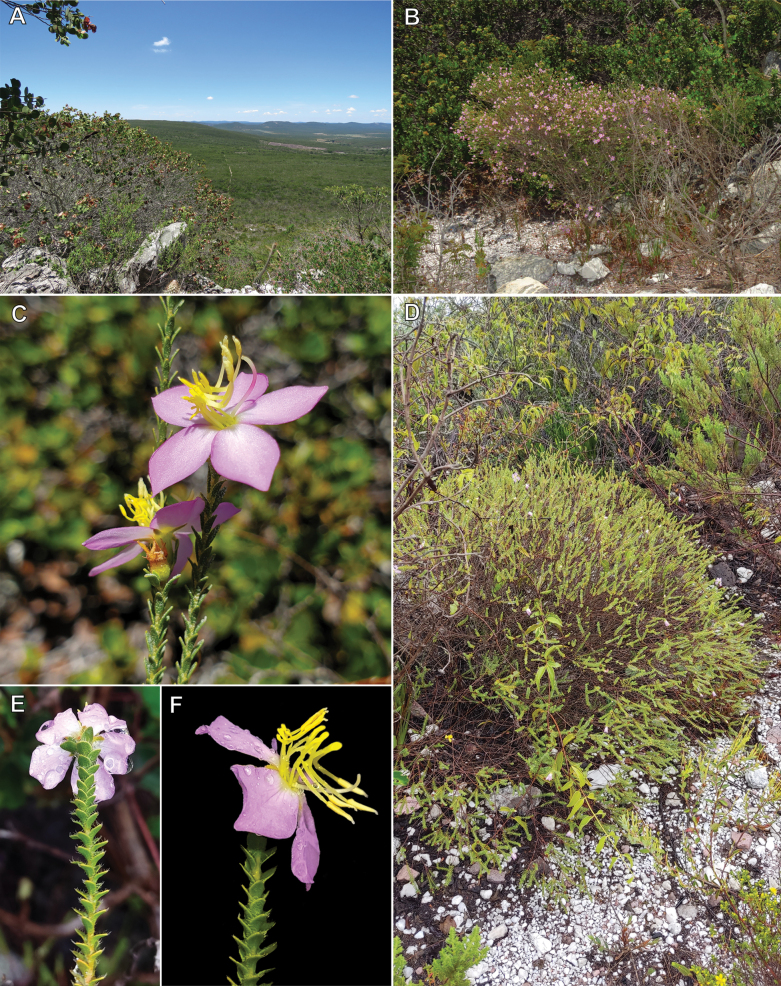
**A.** Landscape at the Serra de Ibitiara, Bahia, Brazil; **B, C.***Microlicia integra*; **B.** Habit; **C.** Flowering branches; **D–F.***Microlicia flavistyla*; **D.** Habit; **E.** Branchlet terminating in a flower at anthesis, view from behind; **F.** Branchlet terminating in a flower at anthesis, in lateral view; Photos: **A–C**, by R. Pacifico; **D–F**, by M.E. Engels.

**Figure 3.**
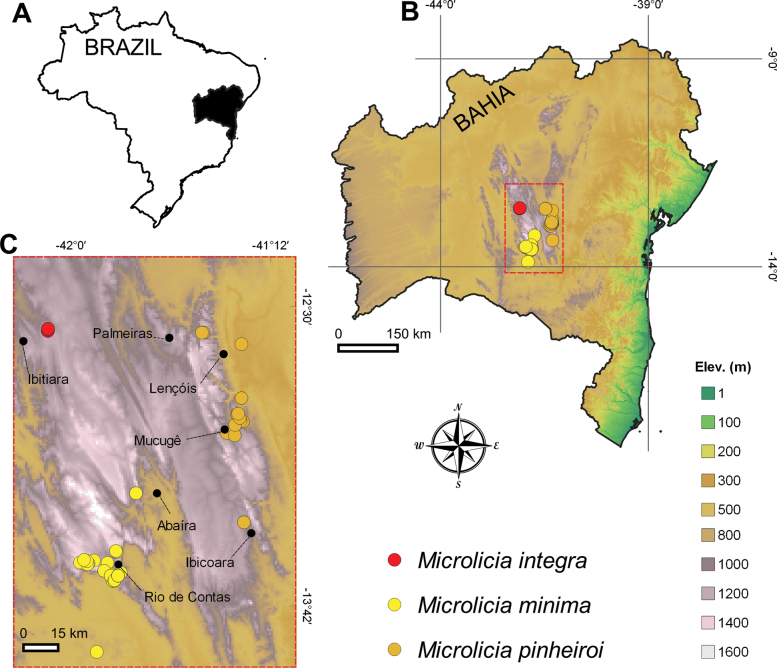
Distribution of *Microlicia integra* and probable relatives. **A.** Brazil with Bahia state highlighted; **B.** Bahia; **C.** Distribution of *M. integra*, *M. minima*, and *M. pinheiroi* on the Chapada Diamantina and surroundings.

Known only from the Serra de Ibitiara (western Bahia; Fig. 3), where it has been collected on campo rupestre between 1,096 and 1,218 m elevation (Fig. 2A), growing on white sand. Collected flowering and fruiting in September and November.

##### Recognition.

Overall, *M. integra* can be recognized by its branches and leaves that are glandular-punctate, 1-nerved, and petiolate; reduced blades 1.8–3.9(–4.5) mm long; triangular calyx lobes, each alternating with an eglandular trichome 0.7–1 mm long (tardily deciduous); petals entirely pink, and dimorphic stamens that are entirely yellow.

Besides *M. minima* (as detailed in the diagnosis), *M. integra* is also morphologically similar to *M. pinheiroi*. The new species differs by its modally shorter petioles, 0.2–0.6 mm long (vs. 0.6–1 mm long in *M. pinheiroi*); leaf blades 1.8–3.9(–4.5) mm long (vs. 4–6 mm long); shorter calyx lobes, 1.6–2.0 mm long (vs. 2.4–2.7 mm long); each calyx lobe alternating with an eglandular trichome ca. 0.7–1 mm long (vs. these trichomes absent); and petals entirely pink (vs. magenta with a cream base). Another possible relative is *M. flavistyla*, from which *M. integra* differs by the leaves and calyx lobes eciliate (vs. ciliate with eglandular trichomes), modally narrower leaves 0.8–1.2(–1.6) mm wide (vs. 1.2–3.3 mm wide), each calyx lobe alternating with a deciduous eglandular trichome (vs. these trichomes absent), and fruiting hypanthia not forming a constricted neck above the capsule summit (vs. neck ca. 0.6 mm long). *Microlicia integra* is clearly allopatric with both *M. minima* and *M. pinheiroi*, with a significant gap between their distributions (Fig. 3). It also appears to be parapatric with *M. flavistyla*, although these species grow in different parts of the same mountain range that crosses the municipality of Ibitiara.

##### Etymology.

The specific epithet *integra* comes from the Latin *integer* (“entire, complete”), referring to the fact that this species bears petals that are entirely pink. In contrast, in its closest relative, *M. minima*, the petals have a cream base.

##### Additional specimens examined (paratypes).

Brazil • Bahia: Ibitiara. Cadeia do Espinhaço, Chapada Diamantina, 12°35'18"S, 42°05'32"W, 1,200 m, 6 September 2022, fl., fr., *M.E. Engels 9981 & D. Liebsch* (UPCB!); • Serra de Ibitiara, Chapada Diamantina, 12°35'32"S, 42°05'30.8"W, 1,201 m, 19 December 2024, fl. fr., *R. Pacifico 737 & I.V. Castro* (CAS!, RB!, UPCB!); • Serra de Ibitiara, Chapada Diamantina, 12°35'17.9"S, 42°05'31.5"W, 1,196 m, 19 December 2024, fl. fr., *R. Pacifico 738 & I.V. Castro* (CAS!, RB!, UPCB!).

#### ﻿ Microlicia flavistyla

Taxon classificationPlantaeMyrtalesMelastomataceae

R.B.Pacifico, Almeda & R.Goldenb. sp. nov.

4422FC6F-E6FD-5C64-993E-E93E7FCCC126

urn:lsid:ipni.org:names:77372163-1

Figs 2D–F, 4

##### Type.

Brazil • Bahia: Ibitiara. Cadeia do Espinhaço, Chapada Diamantina, 12°31'46"S, 42°03'43"W, 1,238 m, 15 Jan 2025, fl., *M.E. Engels & D. Liebsch 12720* (holotype: UPCB barcode UPCB0095340!, isotypes: CAS!, RB!).

**Figure 4.**
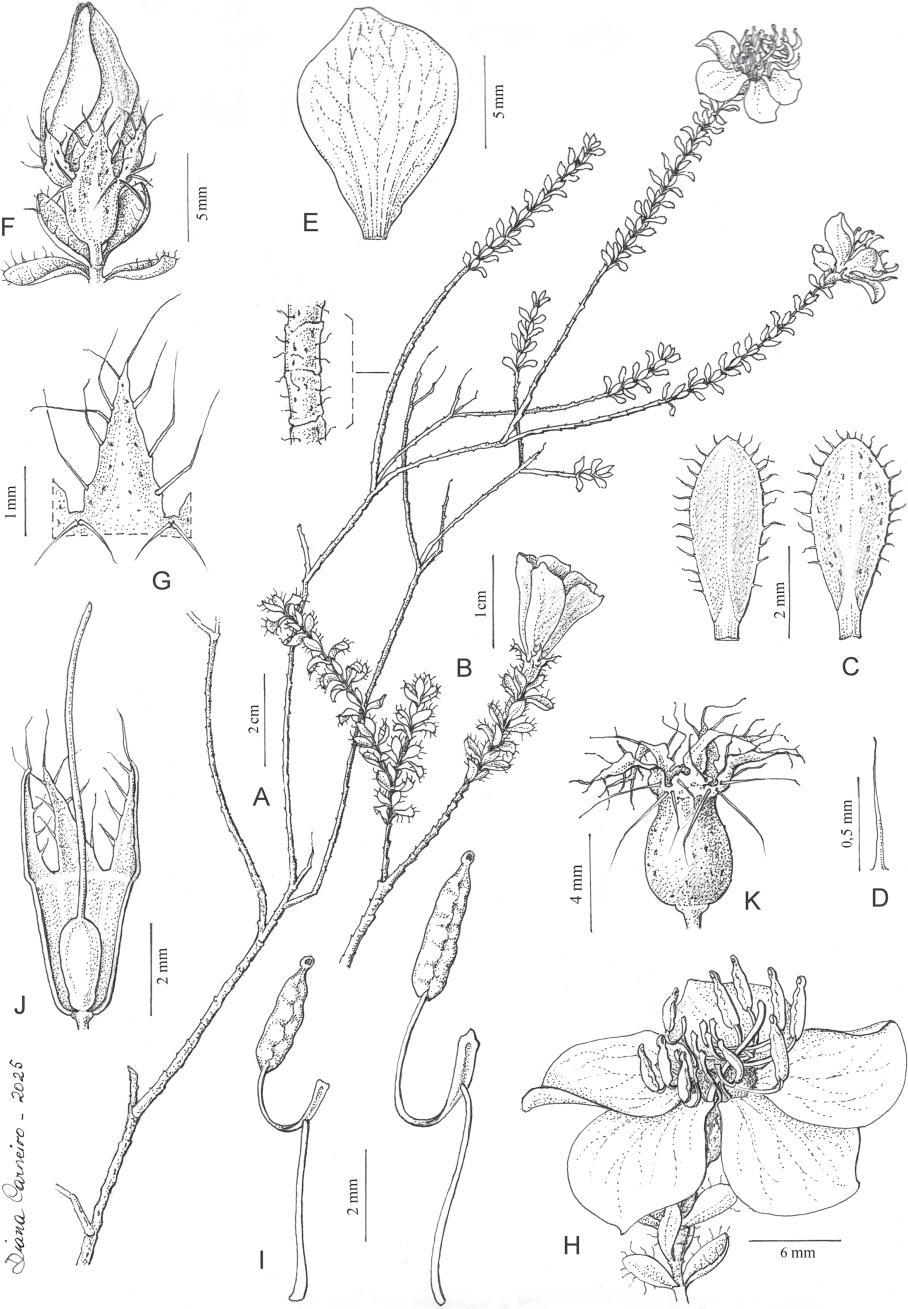
*Microlicia flavistyla*. **A.** Habit; **B.** Branchlet terminating in a flower; **C.** Leaf adaxial (left) and abaxial (right) surfaces; **D.** Detail of leaf margin trichome; **E.** Petal in adaxial view; **F.** Floral bud; **G.** Detail of a calyx lobe; **H.** Flower in lateral view; **I.** Antepetalous (left) and antesepalous (right) stamens; **J.** Gynoecium and enveloping hypanthium at anthesis; **K.** Capsule enveloped by the hypanthium. Drawn from *M.E. Engels 12720 & D. Liebsch*.

##### Diagnosis.

Differs from *M. oligochaeta* Wurdack by the modally shorter leaves, 2.7–4.6 × 1.2–3.3 mm (vs. 4–(5–7) × 3–5 mm in *M. oligochaeta*), that are imbricate to subimbricate (vs. not imbricate), with both surfaces only glandular-punctate (vs. with sparse eglandular trichomes on both surfaces), 1-nerved (vs. 3-nerved), and flowers with shorter calyx lobes, 1.5–1.9 mm long (vs. 2.7–3.2 mm long), and a yellow style (vs. pink or white).

##### Description.

Copiously branched erect shrubs ca. 0.5 m tall. Branchlets light green (when fresh), quadrangular, glandular-punctate, and sparsely covered with eglandular trichomes 0.5–1.2 mm long, the stem angles unwinged. Leaves ascending (when fresh), flat, decussate, ca. 2 times longer than the internodes, on flattened petioles 0.5–0.8 mm long; blades 2.7–4.6 × 1.2–3.3 mm, elliptic to narrowly elliptic, papyraceous, both surfaces vivid green when fresh, pale green when dry, base cuneate to attenuate, margin entire and ciliate with eglandular trichomes 0.5–1.2 mm long, the apex acute to rounded, 1-nerved from the base, venation slightly impressed on the abaxial surface and inconspicuous on the adaxial surface, both surfaces densely glandular-punctate. Flowers (4-)5-merous on inconspicuous pedicels 0.3–0.4 mm long, solitary, terminal, sometimes clustered at the apex of the branchlets. Hypanthia (at anthesis) 2.6–2.8 mm long, 2.0–2.5 mm wide at the torus, yellowish green when fresh, turning brown when dry, campanulate, densely glandular-punctate. Calyx tubes 0.2–0.4 mm long; calyx lobes (at anthesis) 1.5–1.9 mm long (excluding apical trichome), 0.9–1.1 mm wide at the base, triangular, apex acute terminating in an eglandular, persistent trichome ca. 1.0–1.2 mm long, externally glandular-punctate, the margin entire and ciliate with eglandular trichomes ca. 1.8–2.8 mm long, each calyx lobe also alternating with one or a few eglandular, persistent trichomes ca. 1.8–2.8 mm long. Petals 9–11 × 5–7 mm, widely obovate, pink, margin entire, apex bluntly acute to obtuse or emarginate, both surfaces glabrous. Stamens (8)10, dimorphic, entirely yellow; larger (antesepalous) stamens (4)5, filaments 4.2–4.4 mm long, pedoconnectives 6.6–6.9 mm long, appendages 1.3–1.5 mm long, apex truncate, thecae 2.0–2.4 mm long (excluding the rostrum), oblong, yellow, externally corrugated (polysporangiate), rostra 0.3–0.5 mm long, white, the circular pores 0.1–0.3 mm wide, ventrally inclined; smaller (antepetalous) stamens (4)5, filaments 3.2–3.6 mm long, pedoconnectives 2.7–2.9 mm long, appendages 0.9–1.0 mm long, apex truncate, thecae 1.8–2.0 mm long (excluding rostra), oblong, yellow becoming brownish in post-anthesis, externally corrugated (polysporangiate), rostra 0.3–0.5 mm long, the circular pores 0.1–0.2 mm wide, ventrally inclined. Ovaries ca. 2.1 × 1.4 mm, ovoid, superior, glabrous, 3-locular; styles 6.6–7.1 mm long, linear, yellow, stigma punctiform. Loculicidal capsules 3.6–3.8 × 2.5–2.9 mm when mature, ovoid, brownish when dry, initially enveloped by the constricted hypanthium at the apex (neck ca. 0.6 mm long), then tardily rupturing and flaking away with age, the apex not exceeding the torus, dehiscent from the apex to the base, columellas caducous. Seeds not seen.

##### Distribution, habitat, and phenology.

**Figure 5.**
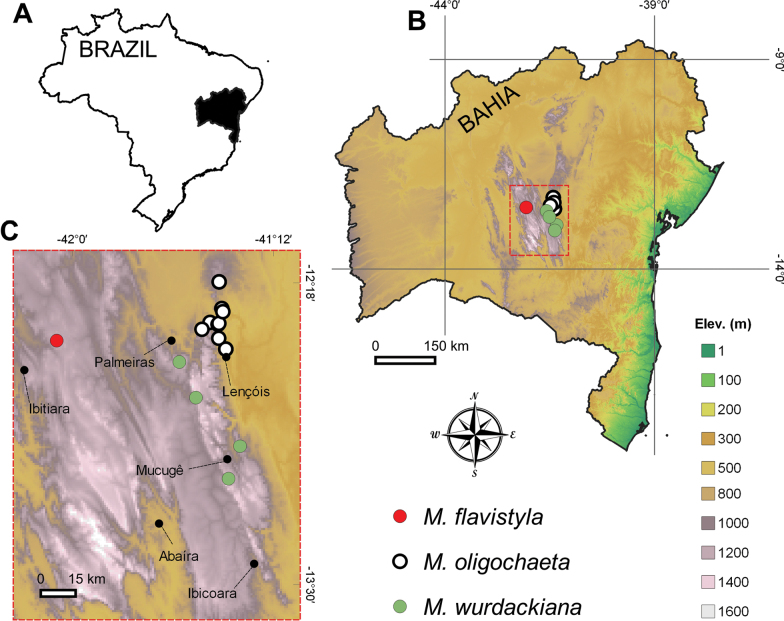
Distribution of *Microlicia flavistyla* and probable relatives. **A.** Brazil with Bahia state highlighted; **B.** Bahia; **C.** Distribution of *M. flavistyla*, *M. oligochaeta*, and *M. wurdackiana* on the Chapada Diamantina.

Like *M. integra*, *M. flavistyla* is known only from the Serra de Ibitiara (western Bahia; Fig. 5), where it has been found on campo rupestre at 1,238 m elevation (Fig. 2A). Collected flowering and fruiting in January.

##### Recognition.

*Microlicia flavistyla* can be recognized by its 1-nerved, petiolate leaves, measuring 2.7–4.6 × 1.2–3.3 mm, with eglandular trichomes 0.5–1.2 mm long restricted to the margins; flowers with pink petals and yellow stamens and style; and polysporangiate anthers.

In addition to its similarities with *M. oligochaeta* (see diagnosis), it also resembles *M. wurdackiana* but differs in that its leaf blades are narrowly elliptic (vs. orbicular-elliptic), glandular-punctate on both surfaces (vs. covered with eglandular trichomes ca. 0.3 mm long on both surfaces), hypanthia only glandular-punctate (vs. densely covered with eglandular trichomes), and calyx lobes ciliate with trichomes 1.8–2.8 mm long (vs. margin of calyx lobes fringed and glandular-punctate). Like *M. integra*, *M. flavistyla* is also evidently allopatric with its morphologically closest relatives (Fig. 5). For a comparison with *M. integra*, see comments under that species.

##### Etymology.

The specific epithet *flavistyla* is derived from the Latin *flavus* (“yellow”) and *stylus* (“style”), referring to the species’ distinctive yellow styles. This contrasts with its closest relatives, in which the styles are pink or white.

##### Additional specimen examined (paratype).

Brazil • Bahia: Ibitiara. Cadeia do Espinhaço, Chapada Diamantina, 12°31'46"S, 42°03'43"W, 1,238 m, 15 January 2025, fr., *M.E. Engels 12717 & D. Liebsch* (CAS!, RB!, UPCB!).

##### Notes on the conservation of *M. integra* and *M. flavistyla*.

Considering that the study area is poorly explored, we opted to classify both species described in this manuscript as Data Deficient (DD) according to the IUCN (2024) criteria. The discovery of these species, along with two other taxa recently described from the campo rupestre areas of Ibitiara (Engels et al. 2023; Gonzaga et al. 2023), highlights the urgent need for systematic floristic inventories, which are essential for an objective assessment of natural resources and the necessity of establishing new conservation units. We believe that given the heterogeneity of natural landscapes in the region, populations of additional plant species may also require protection, particularly in areas of rocky outcrops and campo rupestre habitats developed on white quartz sand substrates. The campo rupestre vegetation in this region seems to be markedly heterogeneous, and although the two new species were found on nearby mountains, they were never recorded co-occurring at the same site.

Initial expeditions to the campo rupestre areas of Ibitiara were motivated by the need to conduct environmental impact studies required for the installation of wind farms in the region. Although wind energy is often promoted as a sustainable alternative, the construction and operation of wind farms can have profound ecological impacts, particularly in sensitive mountainous ecosystems of Brazil. In the northeastern region, a considerable number of wind energy projects have been established at the expense of native vegetation, leading to habitat loss and landscape alteration (Turkovska et al. 2020). As highlighted by Neri et al. (2019) and Paulino et al. (2023), the rapid expansion of wind energy infrastructure must be guided by integrated conservation planning, ensuring that renewable energy development does not come at the cost of biodiversity loss, ecosystem collapse, and social conflicts.

## Supplementary Material

XML Treatment for Microlicia integra

XML Treatment for Microlicia flavistyla

## ﻿Additional information

### Use of AI

No use of AI was reported.

### Author ORCIDs

Ricardo Pacifico https://orcid.org/0000-0001-9566-5344

Frank Almeda https://orcid.org/0000-0001-5091-6875

Mathias Erich Engels https://orcid.org/0000-0003-4332-5166

Renato Goldenberg https://orcid.org/0000-0002-7047-6720

### Data availability

All of the data that support the findings of this study are available in the main text.
